# A Remarkable Wound of the Brain

**Published:** 1882-07

**Authors:** 


### A REMARKABLE WOUND OF THE BRAIN.

The *Lancet* for January, 1882, reports the following wonderful case, which was presented to the Société de Medecine de Paris by M. Dubrisay. A man determined on suicide held in his left hand a dagger about three and one-third inches long and one-third of an inch wide, and placing the point against his skull, struck it several blows with a mallet, believing that he would fall dead at the first blow. To his surprise he felt no pain, and observed no phenomena. He struck the dagger, in all, about a dozen times. When seen, the handle of the dagger was projecting from the skull at the junction of the posterior and middle third, a little to the right of the middle line, and in a transverse position. All of the blade except one-third of an inch was imbedded. The most strenuous efforts were made to remove the dagger, without avail, so tightly was it held in position. Neither did these efforts cause any pain. The man then

*walked* to a coppersmith's, where he was fastened to rings fixed in the ground, and by strong pincers the handle of the dagger was fastened to a chain, which was placed over a cylinder turned by steam power. At the second turn the dagger came out. The patient, during all this time, suffered no pain or inconvenience, and in a few minutes *walked* to a hospital, where he remained in bed for ten days, but without fever or pain. He then returned to his work, and the wound gradually healed. By driving the dagger into the head of a cadaver in the same situation and to the same depth, M. Dubrisay found that, without injuring the superior longitudinal sinus, it had passed into the cerebral substance just behind the ascending parietal convolutions, and thus behind the motor zone ; the point had not reached the base.

